# Genomic landscape of oral squamous cell carcinoma from the southwest coast of Karnataka: insights from FFPE-based next-generation sequencing

**DOI:** 10.3389/fgene.2026.1739925

**Published:** 2026-02-25

**Authors:** Hafeeda Kunhabdulla, Riaz Abdulla, M. Divya Lakshmanan, Rohan Thomas, Devika Jayarajan, Vipul Jain, A. Fahizah, Mohammed S. Mustak, Ranajit Das

**Affiliations:** 1 Department of Oral Pathology, Yenepoya Dental College, Yenepoya (Deemed to be University), Mangalore, Karnataka, India; 2 Division of Cancer Research and Therapeutics, Yenepoya Research Centre, Yenepoya (Deemed to be University), Mangalore, India; 3 Department of Surgical Oncology, Zulekha Yenepoya Institute of Oncology, Yenepoya (Deemed to be University), Mangalore, India; 4 Department of Biomaterials and Research, Yenepoya Dental College, Yenepoya (Deemed to be University), Mangalore, Karnataka, India; 5 Department of Applied Zoology, Mangalore University, Mangalore, Karnataka, India; 6 Division of Data Analytics, Bioinformatics and Structural Biology (DABS), Yenepoya Research Centre, Yenepoya (Deemed to be University), Mangalore, India

**Keywords:** FFPE, genomic profiling, India, Karnataka, mutational landscape, next-generation sequencing, oral squamous cell carcinoma

## Abstract

**Background:**

Oral squamous cell carcinoma (OSCC) remains a major health burden in India, yet region-specific genomic data are limited. This study aimed to characterize the mutational landscape of OSCC patients from the southwest coast of Karnataka using FFPE tissues and assess potential clinical correlations.

**Methods:**

Whole-exome sequencing was performed on tumor and adjacent normal FFPE samples from 21 OSCC patients. Variants were annotated using multiple clinical databases, and stratified analyses were conducted across clinicopathological parameters including age, sex, tumor site, and TNM stage.

**Results:**

We identified extensive inter-patient variability in clinically relevant mutations, with intronic and missense variants being most frequent. A core set of 21 genes including *ABCB1, CD44, IL6, PADI2*, and *VKORC1*—carried pathogenic or drug-response variants in all patients. Ten tumor-exclusive mutations were observed, including *TLR1* rs5743618, present in 100% of tumors. Pathway and network analyses highlighted enrichment in p53 signaling, immune pathways, and platinum-drug resistance. Stratified analyses showed no significant differences in mutation burden across TNM stages (Kruskal–Wallis p = 0.952), nodal status (p = 0.460), age, or sex. Polygenic risk score estimation revealed that 15 of 21 patients belonged to the highest-risk quartile, suggesting strong inherited susceptibility.

**Conclusion:**

FFPE-based genomic profiling successfully captured key OSCC-associated alterations and revealed region-specific mutation signatures. The predominance of germline and pharmacogenomic variants and strong PRS enrichment underscore the potential of incorporating hereditary risk assessment and targeted therapy selection into OSCC management strategies in this population.

## 1 Introduction

Head and neck squamous cell carcinoma (HNSCC) arises from the mucosal lining of the upper aerodigestive tract, including the larynx, hypopharynx, tonsil, oropharynx, and oral cavity. Oral squamous cell carcinoma (OSCC), the most common subtype of HNSCC, originates from the epithelial and mucosal surfaces of the tongue, buccal and labial mucosa, alveolar ridge, palate, and other oral cavity regions (Ranganathan et al., 2023; Paul et al., 2025). According to GLOBOCAN 2022 estimates, cancers of the lip and oral cavity remain a significant health burden in India, accounting for approximately 390,000 new cases and over 188,000 deaths annually, with OSCC representing more than 90% of all oral malignancies (Johnson et al., 2020). These alarming statistics underscore the urgent need for improved prevention, early detection, and personalized treatment strategies.

Although tobacco and alcohol are well-established risk factors for OSCC, disease development and treatment response are influenced by complex interactions between environmental exposures and host genetic susceptibility (Tan et al., 2023; Kunhabdulla et al., 2024). Cases in individuals without any known habits further highlight the contribution of genetic factors to oral carcinogenesis. Variation in driver mutations and dysregulated pathways between populations suggests the importance of region-specific genomic profiling to inform tailored therapies (Paul et al., 2025; Abdulla et al., 2023; Uddin et al., 2022; Mishra et al., 2023).

Next-generation sequencing (NGS) enables comprehensive detection of somatic alterations, providing opportunities to identify clinically actionable biomarkers (Leemans et al., 2018). Genotypic analysis of Long noncoding RNAs (lncRNA) such as ANRIL and H19 and their genetic polymorphisms have recently been associated with OSCC (Ghapanchi et al., 2020; Amiri et al., 2023; Salah et al., 2024). However, reliance on fresh tumor biopsies limits routine implementation. Formalin-fixed paraffin-embedded (FFPE) tissues offer a practical alternative for genomic studies, allowing integration of mutational profiles with long-term clinical outcomes (Gayathri et al., 2022; Henshall et al., 2023; Mäki-Nevala et al., 2021). Despite several genomic investigations in India, primarily involving North Indian patients or focusing on targeted cancer gene panels (Goel et al., 2021; Tandon et al., 2022; India Project Team of the International Cancer Genome Consortium, 2013; Patel et al., 2021; Paul et al., 2025) there remains a lack of whole-exome studies from South Indian populations, particularly those along the southwest coast of Karnataka where OSCC is highly prevalent.

To address this critical knowledge gap, the present study performed whole-exome sequencing of paired tumor and normal FFPE samples from OSCC patients in coastal Karnataka. By characterizing somatic and germline variants and correlating them with clinicopathological features, this work aims to identify population-relevant genomic alterations with potential diagnostic, prognostic, and therapeutic significance.

## 2 Methods

### 2.1 Sample collections

Forty-eight FFPE tissue samples from patients diagnosed with OSCC, including both tumor and adjacent normal tissues from the southwest coast of India, were collected from Yenepoya Dental College and Hospital, Deralakatte within a duration of 2022–2024. The study received approval from the institutional ethical review boards of Yenepoya medical College and hospital ethics committee (Protocol No: YEC-1/2023/131). After quality control measures 13 samples were discarded. The remaining 35 samples were employed for the downstream analysis.

### 2.2 Whole exome sequencing

Whole exome sequencing (WES) was used to identify the mutation. Tumor and adjacent normal tissues of the OSCC patients were collected from the Yenepoya Dental College and Hospital for the mutational profiling. Out of the 35 samples 14 pairs (N = 28) were associated with the same patient (14 normal adjacent tissues as control and 14 OSCC tumor tissues as cases) from 14 individuals were employed in this study. Remaining seven were single tissue samples (five only tumor tissues and two only normal tissues). Total number of tumor tissues were 19 and total number of normal tissues were 16. The clinical details of the recruited samples is summarized in Table 1.

**TABLE 1:** Clinical details of the recruited samples.

HPE No	Original TNM	Medical history	Age	Sex	Site	Simplified stage	Mutational profiling
*10026E/23*	PT2N0	Radio therapy done before surgery	44	Male	Right lateral border of tongue	Stage II	Only normal tissue
*10175R2/23*	PT2N0Mx	Chemo done before surgery	58	Female	Tongue left lateral border	Stage II	None
*4384A/23*	PT2N0	No relevant history	44	Male	Right buccal mucosa	Stage II	Only tumor tissue
*4741H/23*	PT1N0	No relevant history	80	Male	Left upper lip	Stage I	Only tumor tissue
*4968E/23*	PT2N0	Radio therapy done before surgery	49	Male	Left lateral border of tongue	Stage II	Both tumor and normal
*4975/23*	PT2N2b	No relevant history	56	Female	Right retromolar trigone	Stage IV	Both tumor and normal
*5058F/23*	PT4aN1	Hyperthyroidism under medication, surgery post chemo	47	Female	Left lower alveolus	Stage IV	Only tumor tissue
*5103G/23*	PT4aNx	Radio therapy, cancer recurrence, chemo done	53	Male	Right upper alveolus	Stage IV	Only tumor tissue
*5212E/23*	PT2N2b	No relevant history	41	Male	Right buccal mucosa	Stage IV	Both tumor and normal
*5286G/23*	PT4ANb2	Hypertension under medication	46	Female	Right buccal mucosa	Stage IV	Both tumor and normal
*5288G/23*	PT4AN3b	Radio therapy done before surgery	75	Male	Left lower alveolus	Stage IV	Both tumor and normal
*5521F/23*	PT2N2b	Chemo done before surgery	41	Male	Right buccal mucosa	Stage IV	Both tumor and normal
*6331D/23*	PT1N0	Diabetes, hypertension	55	Female	Left buccal mucosa	Stage I	Both tumor and normal
*6627B/23*	PT2N1	Cisplatin poor tolerance	59	Female	Right buccal mucosa extending to upper alveolus	Stage III	Only normal tissue
*6702E/23*	PT2N0	No relevant history	76	Female	Right lower alveolus + marginal mandibulectomy	Stage II	Only tumor tissue
*6750C/23*	PT3N1	Diaphragmatic palsy, adjuvant radio therapy	72	Male	Right lateral border of tongue	Stage III	Both tumor and normal
*7736J/23*	PT4aN2b	No relevant history	80	Female	Left buccal mucosa	Stage IV	Both tumor and normal
*8412E/23*	PT2N0	Diabetic under ayurvedic medication	58	Male	Right buccal mucosa	Stage II	Both tumor and normal
*9392E/23*	PT2N2bMx	Radio therapy done post-surgery	52	Male	Lateral border of tongue	Stage IV	None
*9465C/23*	PT1N1	No relevant history	55	Female	Right lower lip	Stage III	Both tumor and normal
*9472C/23*	PT2N1	No relevant history	69	Male	Left buccal mucosa	Stage III	Both tumor and normal
*9640E/23*	PT2N0	Hypertension under medication	82	Male	Lower lip	Stage II	Both tumor and normal
*9653E/23*	PT3N0	No relevant history	75	Male	Left angle of mouth + right buccal mucosa	Stage III	None
*9675D/23*	PT2N0	No relevant history	50	Male	Left lateral border of tongue	Stage II	Both tumor and normal

Whole Exome Sequencing (WES) libraries were prepared using Twist Exome 2.0 kit. Genomic DNA was extracted from the tissue samples, and samples with a DNA concentration of ≥20 ng/μL were selected for WES. All samples were sequenced on Illumina NovaSeq6000 system. The raw paired-end sequencing reads (PE150) generated from FFPE samples were initially processed for adapter removal and quality filtering to obtain high-quality trimmed reads. These processed reads were then mapped to the *Homo sapiens* reference genome GRCh37 using the Burrows–Wheeler Aligner (BWA) with default parameters (Li and Durbin, 2009). Variant calling followed standard best-practices workflows, where aligned BAM files were refined and variants were identified. The resulting VCF files were subsequently annotated using Ensembl’s Variant Effect Predictor (VEP) (Hunt et al., 2022) to assign functional consequences to each variant. A matched tumor–normal comparison strategy was used to distinguish somatic mutations from germline variants, emphasizing those alterations potentially driving tumorigenesis. All processed datasets comprised FASTQ, BAM, VCF, and annotated output files, as delivered in the sequencing report from NCGM.

### 2.3 Discovery of mutational landscape

To annotate the detected genetic variants, multiple clinically relevant databases and prediction tools were employed to classify variant types and assess their pathogenic potential. Tumor tissue was compared with adjacent normal tissue to distinguish somatic alterations from germline variants, particularly those implicated in tumor development. The dbSNP database was used as a comprehensive resource for cataloging genetic variations across different species and providing detailed information on each variant. ClinVar, a specialized repository for clinically important genetic alterations (Landrum et al., 2018), was utilized to identify variants classified as pathogenic or likely pathogenic. Additionally, integrated computational tools, PolyPhen-2 (Adzhubei et al., 2010) were applied to predict the functional consequences of the mutations. The OMIM® database was further consulted to determine any known links between the identified variants and genetic disorders.

### 2.4 Polygenic risk score (PRS) estimation

To estimate PRS, we merged exome-wide data from 21 OSCC patients with genome-wide data of 204 South Asian individuals from our lab database using PLINK v1.9. The merged. bed file assessed 5,341 SNPs. PRSice2 (Choi et al., 2019) was employed to estimate PRS of oral cancer for the 21 OSCC patients and other individuals present in the merged dataset. Summary statistics from MVP_R4.1000G_AGR.Phe_145_2. META.GIA.dbGaP.txt was downloaded through GWAS catalogue and was employed as the reference (--base) data during PRS estimation. Notably, the reference file compared 2,203 oral cancer patients against 570,152 controls, assessing 17, 701, 051 SNPs. The test samples were divided into PRS quartiles; the lowest quartile (bottom 25%) was considered as reference. All subsequent PRS groups were considered to have an increased risk of developing oral cancer compared to the reference quartile.

## 3 Results

### 3.1 Patient details

This retrospective study analysed 48 patients with histopathologically confirmed Oral squamous cell carcinoma (OSCC) who underwent surgical resection at Yenepoya Medical College Hospital, Mangalore. Within a duration of (2022–2025) Formalin-fixed paraffin-embedded (FFPE) tissue samples were retrieved from the archives of Yenepoya Dental College Hospital after OSCC diagnosis confirmation. Collected clinicopathological data included (Table 2) age, gender, surgery date, primary tumor site, AJCC 8th edition TNM stage, tumor size, and tobacco use history.

**TABLE 2:** Molecular correlates of clinicopathological variables in oral squamous cell carcinoma.

Variable	Category	Frequency (n = 24)	Percentage (%)
Age (years)	<45	7	29.2
​	≥45	17	70.8
Sex	Male	16	66.7
​	Female	8	33.3
Habits	Tobacco chewing	20	83.3
​	Tobacco smoking	6	25.0
​	Alcohol consumption	4	16.7
​	None	0	0.0
Site of lesion	Buccal mucosa (left & right)	7	29.2
​	Lateral border of tongue	7	29.2
​	Lower alveolus + RMT lesion	5	20.8
​	Upper alveolus	1	4.2
​	Upper lip	1	4.2
​	Angle of mouth	1	4.2
TNM staging	Stage I	7	29.2
​	Stage II	7	29.2
​	Stage III	1	4.2
​	Stage IV	9	37.5
Broder’s classification	SCC G1	2	8.3
​	SCC G2	22	91.7
Location	South Karnataka	17	70.8
​	North Karnataka	5	20.8
​	Kerala	2	8.3
Language	Kannada	9	37.5
​	Malayalam	2	8.3
​	Hindi/Tulu (others)	3	12.5

The numbers in the “Percentage” column, shown in parentheses, represent the percentage of the total observations that fall into each category. Abbreviations Used: OSCC: Oral Squamous Cell Carcinoma RMT: retro molar trigone, TNM: tumor, Node, Metastasis (a cancer staging system), SCCG1: Squamous Cell Carcinoma Grade 1, SCCG2: Squamous Cell Carcinoma Grade 2.

The cohort comprised 18 (37.5%) females and 30 (62.5%) males. The mean age was 54.80 years (SD = 10.1), with a median of 55. The most common primary tumor site was the buccal mucosa (n = [10], (41.6%), followed by the [lateral border of tongue] (n = [6] % Choi et al., 2019) and the [lower alveolus and lower lip] (n = [5,3], [20.8,12.5] %)., TNM stage distribution, mean tumor size. The most common site was the buccal mucosa presenting as moderately differentiated OSCC (Table 3).

**TABLE 3:** Distribution of tumor stages (pT1-pT4a) across oral cavity subsites.

*Site*	*pT1*	*pT2*	*pT3*	*pT4a*	*Total*
Buccal mucosa	1	3	3	3	10
Lip	1	1	0	0	2
Alveolus/GBS/RMT lesion	1	2	0	4	7
Tongue	0	4	1	0	5
Total	**3**	**10**	**4**	**7**	**24**

Bold values indicate pathological tumor (pT) staging categories, where “p” denotes pathological staging and T1–T4a represent tumor stage.

The table illustrates how tumor stage (pT1-pT4a) varies across different subsites in the oral cavity, highlighting the distribution of tumor advancement at diagnosis for each location. pT (Pathological Tumor): This refers to the size and extent of the primary tumor, determined after surgical removal and pathological examination. pT staging describes the size and local extent of the primary tumor after pathological examination. pT1-pT4a indicate increasing tumor size and/or invasion into nearby tissues, with specific measurements and involvement of adjacent structures defining each stage.

### 3.2 Genetic variation among the recruited patients

ClinVar classification system was used to determine clinically relevant genetic variants (SNVs and DELINs). Variants designated with classifiers such as pathogenic, likely pathogenic, risk allele, drug response, association, likely association, and protective were used for downstream analysis. We found discernible variation among the 19 recruited patients with tumor tissue samples in terms of the total number of clinically relevant mutations identified. While patient no.1 had the lowest number of (N = 132) clinically relevant variants, patient no.4 had the highest (N = 197).

**FIGURE 1:**
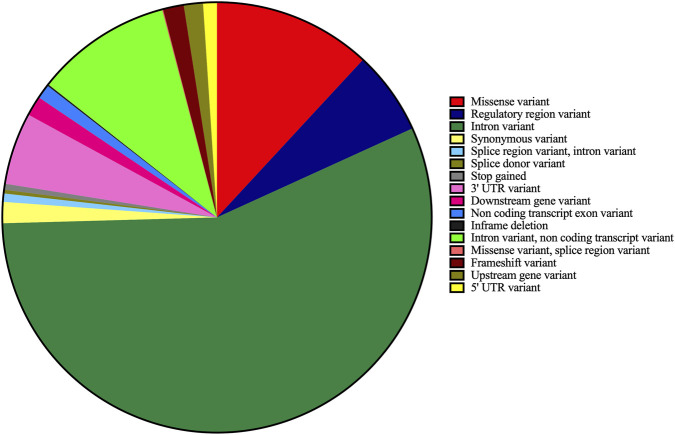
Distribution of clinically relevant variant types identified in OSCC tumor samples. Pie chart illustrating the distribution of clinically relevant genetic variant types identified in tumor tissues of OSCC patients. Intronic variants constituted the majority ( _~_ 56%), followed by missense variants ( _~_ 12%) and non-coding transcript variants (∼10%). Other categories included regulatory region variants, splice region variants, synonymous variants, frameshift variants, and stop-gained mutations, each contributing smaller proportions. Stop-gained variants were observed at very low frequencies (<1%). Percentages represent the average proportion across tumor samples.

Among the various types of genetic variants identified, the intronic variants comprised of 56% ± 7% of all identified variants, followed by missense variants (12% ± 1%) and non-coding transcript variants (10% ± 2%). Stop-gained mutations were found to be present in very low frequencies (<1%) (Figure 1).

The origin of the mutation was determined by comparing the mutations identified in the tumor tissue and those identified in the nearby normal tissue. Based on the origin of the mutations, out of an average of 171 clinically relevant mutations (range: 150–197), most were germline (93.3% ± 3.9%), and 6.7% ± 3.9% were somatic.

**FIGURE 2:**
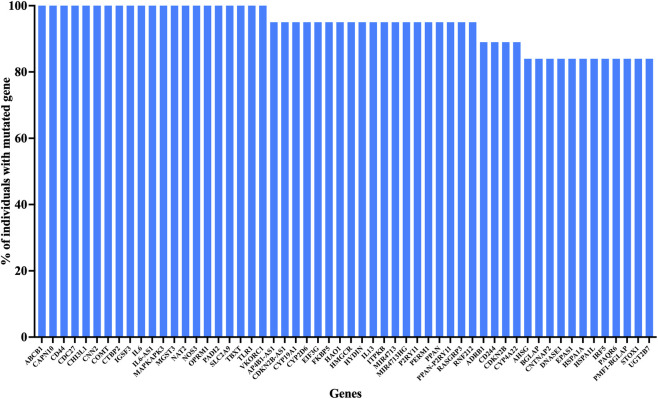
High-frequency mutated genes Identified in OSCC tumor samples. Bar graph showing the percentage of OSCC patients harboring clinically relevant mutations in genes mutated in ≥80% of tumor samples. A total of 56 genes met this threshold, with 21 genes (including ABCB1, CD44, IL6, PADI2, TLR1, and VKORC1) exhibiting mutations in 100% of patients. The y-axis represents the percentage of individuals carrying mutations in each gene, and genes are plotted along the x-axis.

**FIGURE 3:**
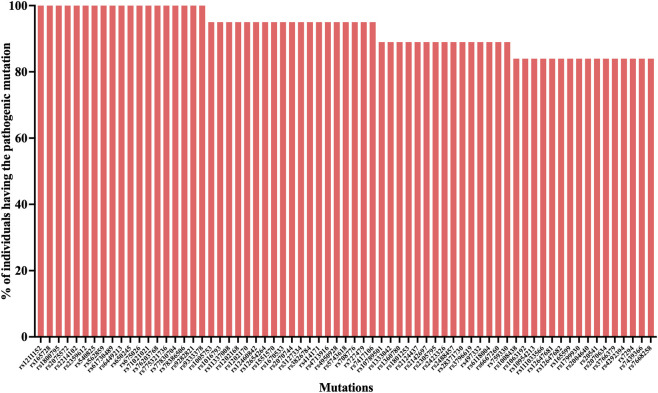
High-frequency clinically relevant mutations in OSCC tumor samples. Bar graph depicting clinically relevant mutations present in ≥80% of OSCC tumor samples. A total of 69 mutations met this threshold, of which 19 were identified in all recruited patients. The y-axis indicates the percentage of individuals harboring each mutation, and specific mutation identifiers (rsIDs) are shown on the x-axis.

We identified 56 genes with at least one clinically relevant mutation in at least 80% of the recruited patients in the tumor tissue (Figure 2). Interestingly, mutations in 21 of these genes (*ABCB1, CAPN10, CD44, CDC27, CHI3L1, CNN2, COMT, CTBP2, IGSF3, IL6, IL6-AS1, MAPKAPK3, MGST3, NAT2, NOS3, OPRM1, PADI2, SLC2A9, TBXT, TLR1* and *VKORC1*) were identified in all recruited patients. We identified 69 clinically relevant mutations that are present in at least 80% of the recruited patients in the tumor tissue (Figure 3). Among them, 19 mutations were identified among all recruited patients. The clinically relevant mutations that were identified in the tumor of at least 50% recruited patients are summarized in Supplementary Table S1.

We note here that, in The Cancer Genome Atlas (TCGA) - Head and Neck Squamous Cell Carcinoma (TCGA-HNSCC) dataset, recurrent somatic alterations are largely confined to well-established oncogenic drivers, including *TP53, PIK3CA,* and *FAT1*. In contrast, most genes found to be mutated in ≥50% of the recruited patients in our cohort predominantly encompassing drug-metabolizing enzymes, receptor genes, and immune-related regulators.

### 3.3 Identification of exclusively tumor-specific mutations

We identified exclusively tumor specific mutations by comparing the mutations identified in the tumor tissue with those identified in the nearby non-tumor normal tissue (N = 14 pairs). We identified ten mutations that were exclusively tumor specific, and present in at least 20% of the recruited patients (Table 4). Among them a single nucleotide variant rs5743618 of *TLR1*was found to be present in the tumor tissue of all recruited patients, followed by rs1805010 (intergenic) and rs546905091 (in *PRKDC*) that were present among 46% recruited patients. Notably, while the minor allele frequency (MAF) of rs5743618 is low (0.25) among European descents, it is very common among Asians (0.89 among South Asians and 0.99 among East Asians), underscoring the importance of multiple reference genomes to understand the global mutational landscapes (Karczewski et al., 2020). On the contrary, the pathogenic insertion mutation rs546905091 is extremely rare among South Asians (MAF = 0.0076), and this highlights its potential role in cancer (Auton et al., 2015). Interestingly, rs1805010, while designated as a pathogenic SNV in ClinVar, is also quite common among South Asians (MAF = 0.44) and also globally, indicating the importance of understanding the role of common genetic variants in the development of multifactorial disorders such as cancers.

**TABLE 4:** Mutations identified exclusively in the tumor tissue of at least 20% recruited patients.

Mutation	Frequency (%)^*^	Chromosome	Position	Variant class	Variant type	Gene	ClinVar_CLNSIG	Amino_acids	Codons	Comparison with TCGA-HNSCC
rs5743618	100	4	38798648	Missense variant	SNV	*TLR1*	Uncertain risk allele	S/I	aGc/aTc	No recurrent somatic calls in TCGA-HNSC (≈0% in public somatic lists)
rs1805010	46	16	27356203	Regulatory region variant	SNV	*IL4R*	Pathogenic	-	-	Not reported as recurrent somatic in TCGA-HNSC.
rs546905091	46	8	48844056	Intron variant	insertion	*PRKDC*	Pathogenic	-	-	No evidence of this rsID as a recurrent somatic event in TCGA-HNSC.
rs4337789	31	4	69973044	Intron variant	SNV	*UGT2B7*	Drug response	-	-	Not observed as a recurrent somatic hotspot in TCGA-HNSC.
rs752940775	31	15	93540315	Frameshift variant	insertion	*CHD2*	Pathogenic	E/EX	gaa/gAaa	Not a TCGA-HNSC somatic hotspot
rs1143634	23	2	113590390	Synonymous variant	SNV	*IL1B*	Affects	F	ttC/ttT	Not a TCGA-HNSC somatic hotspot
rs5744168	23	1	223285200	Stop gained	SNV	*TLR5*	Risk factor	R/*	Cga/Tga	No recurrent TCGA-HNSC somatic evidence
rs61737946	23	22	42523505	Missense variant	SNV	*CYP2D6*	Drug response	G/S	Ggt/Agt	Very rare somatic hits in TCGA
rs764008859	23	13	77581682	Frameshift variant	deletion	*FBXL3*	Pathogenic	L/X	tTa/ta	Not a recurrent TCGA-HNSC somatic variant
rs7809208	23	7	87214275	Intron variant	SNV	*ABCB1*	Drug response	-	-	Not a recurrent TCGA-HNSC somatic variant

Further, we systematically queried TCGA and focused on tumor-specific somatic variants, which revealed either very low or an absence of such mutations for these genes (Table 4). This disparity highlights a distinct mutational landscape in our cohort relative to TCGA, potentially reflecting cohort-specific biological features or differences in variant origin and detection.

Subsequently, a patient-wise comparison was performed to evaluate the overlap between mutations identified in this cohort and the most frequent canonical somatic drivers described in the TCGA-HNSCC dataset (Table 5). Of the established TCGA-HNSCC driver genes assessed, *TP53* was the most frequently detected, present in five of 21 patients, followed by *CDKN2A*, identified in three patients. In addition, *EGFR*, a gene predominantly altered through copy-number amplification in TCGA-HNSCC, was observed in a single patient. Notably, several other genes that are commonly reported as recurrently mutated in TCGA-HNSCC: including *FAT1, NOTCH1, KMT2D, NSD1, CASP8,* and *HRAS* were not detected in this cohort. Collectively, these findings indicate a restricted overlap with the canonical TCGA-HNSCC mutational spectrum, suggesting that the genomic architecture of this cohort is shaped by distinct, potentially population- or exposure-specific factors rather than reflecting the dominant driver landscape reported in large, predominantly Western TCGA datasets.

**TABLE 5:** Presence of canonical TCGA–HNSCC driver gene alterations across the study cohort.

Genes commonly mentioned in TCGA- HNSCC	Recruited patients (N = 21)	TCGA relevance
*TP53*	5	Core TCGA-HNSCC driver
*CDKN2A*	3	Core TCGA-HNSCC driver
*EGFR*	1	TCGA-HNSCC amplification-driven gene
Others (*FAT1, NOTCH1, KMT2D, NSD1, CASP8, HRAS, FBXW7, CCND1, MYC, PIK3CA*)	0	-

### 3.4 Stratified analyses

Stratified analyses were performed to investigate whether the mutation landscape varied across key clinical and demographic dimensions, including age, sex, primary tumor site and pathological TNM stage (Supplementary Table S2). Comparison of mutation burden across disease stages using the Kruskal–Wallis test did not reveal statistically significant differences (p = 0.952), and post-hoc Dunn’s multiple comparison tests further confirmed that mutation burden did not vary significantly between any individual stage pairs (all adjusted p = 1.0). Likewise, comparison between node-negative and node-positive tumors (N0 vs. N+), used as a proxy for metastatic potential, demonstrated no significant difference in mutation burden (Wilcoxon rank-sum test p = 0.460). Subgroup analyses based on age category and sex also showed no discernible variation in mutational load across these strata. Fisher’s exact tests for each gene similarly did not identify any individual variant or gene disproportionately associated with advanced vs. early disease after false-discovery rate correction. Collectively, these results suggest that in this OSCC cohort, comprising patients with predominantly uniform gene panels, the global mutation burden and the presence of the pharmacogenomically associated variants assessed here do not appear to be strongly driven by TNM stage progression or major demographic parameters. Further, our results indicate that additional molecular mechanisms, such as copy-number changes, epigenetic dysregulation, immune microenvironmental factors, or environmental exposures, may have a more prominent role in OSCC clinical heterogeneity.

### 3.5 Comparison of high-frequency mutation genes with dbGENVOC database

Out of the 40 most frequent genes identified in the tumor tissue that have at least one mutation in at least 95% of the recruited patients, 38 were found to be present in dbGENVOC, the comprehensive database with clinically pertinent somatic and germline variation data originating from >100 Indian oral cancer patients (Pradhan et al., 2021). This indicates that clinically relevant mutations identified in *TBXT* and *PERM1*, present in 100% and 95% recruited patients are novel mutations associated with OSCC patients from southwest India. Novel mutations in *XRCC1* and *ITPKB*, previously identified by our team (Paul et al., 2025) was found among ∼80% and 95% of the recruited patients respectively, further highlighting the importance of understanding local mutational landscape. Notably, the missense variant rs25487 in *XRCC1* has been associated with chemotherapy response. Among the 21 genes with exclusively tumor specific mutations in at least two recruited patients, 18 were found in dbGENVOC and two (*CHROMR* and *DNAAF4*) were novel. *CHROMR* (Cholesterol Induced Regulator of Metabolism RNA) (Barriocanal et al., 2022) is a long noncoding RNA (lncRNA) gene that is a key arbitrator of antiviral innate immune signaling in human while *DNAAF4* (Dynein axonemal assembly factor 4) is involved in the preassembly of the multi-subunit dynein protein, which is essential for the proper functioning of cilia and flagella.

### 3.6 GO annotation of genes with exclusively tumor specific mutations

Gene ontology analysis was performed on the 21genes with exclusively tumor specific mutations in at least two recruited patients revealed. The biological processes (BP) of these genes are related to B cell lineage commitment, Replicative and Cellular senescence, Hematopoietic stem cell differentiation, Protein destabilization, rRNA processing, Cell cycle G1/S phase transition, and Regulation of G1/S transition of mitotic cell cycle. The main cellular components (CC) of these genes involve Striated muscle dense body, Cajal body, Nucleoid, Z-disc, I-band, Sarcomere, and Spliceosomal complex. The major molecular functions (MF) associated with these genes are Mitochondrial promoter sequence-specific DNA binding, Biotin transmembrane transporter activity, MDM2/MDM4 family protein binding, Cyclin-dependent protein serine/threonine kinase inhibitor activity, Disordered domain specific binding, Transcription coactivator binding, Protein kinase inhibitor activity and P53 binding.

### 3.7 Pathway analysis

**FIGURE 4:**
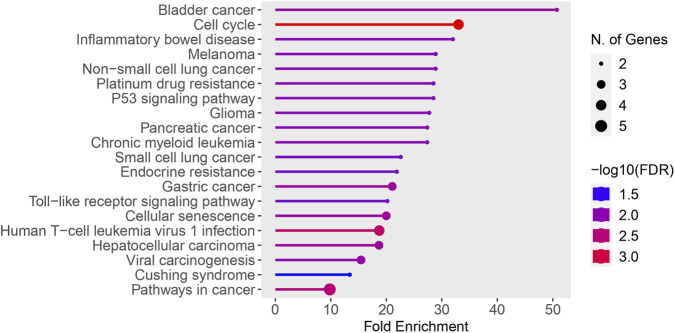
KEGG pathway enrichment analysis of genes with exclusively tumor-specific mutations. Bubble plot representing KEGG pathway enrichment analysis of 21 genes harboring exclusively tumor-specific mutations in at least two patients. Pathways are ranked based on fold enrichment. Bubble size corresponds to the number of genes enriched in each pathway, and color intensity represents statistical significance (-log10 FDR). Significant enrichment was observed in cancer-related pathways including p53 signaling, pathways in cancer, platinum drug resistance, melanoma, bladder cancer, and chronic myeloid leukemia.

**FIGURE 5:**
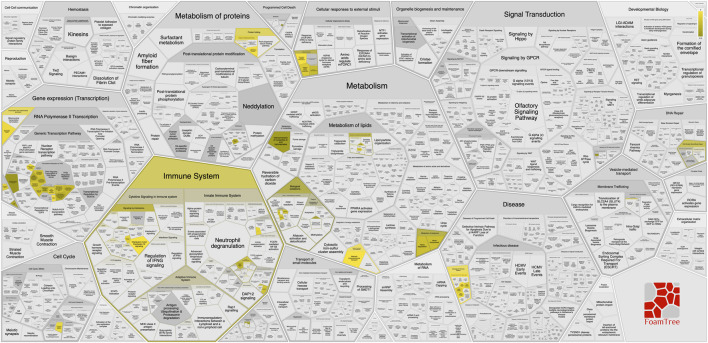
Reactome-based functional classification of genes with tumor-specific mutations. FoamTree visualization illustrating reactome pathway enrichment of genes with exclusively tumor-specific mutations. Major functional categories include immune system, signal transduction, metabolism, disease, and programmed cell death/senescence. Highlighted regions indicate significantly enriched pathways, demonstrating involvement in immune signaling, interleukin pathways, and cancer-associated biological processes.

The 21genes with exclusively tumor specific mutations in at least two recruited patients were analyzed for KEGG enrichment. The results revealed significant enrichment of these genes in various cancers and cancer-related pathways such as Pathways in cancer (hsa05200), Bladder cancer (hsa05219), Melanoma (hsa05218), Non-small cell lung cancer (hsa05223), p53 signaling pathway (hsa04115), Pancreatic cancer (hsa05212), Chronic myeloid leukemia (hsa05220) and Gastric cancer (hsa05226), as well as Platinum drug resistance (hsa01524) (Kanehisa et al., 2021; Gene Ontology Consortium, 2021) (Figure 4). In congruence with KEGG, Reactome pathway analysis revealed that these 21 genes are significantly associated with programmed cell death and senescence. This analysis also identified enrichment of these genes in immune system related pathways such as Interleukin signalling and disorders associated with immune system (Figure 5).

### 3.8 Protein-protein interaction (PPI) network complex

**FIGURE 6:**
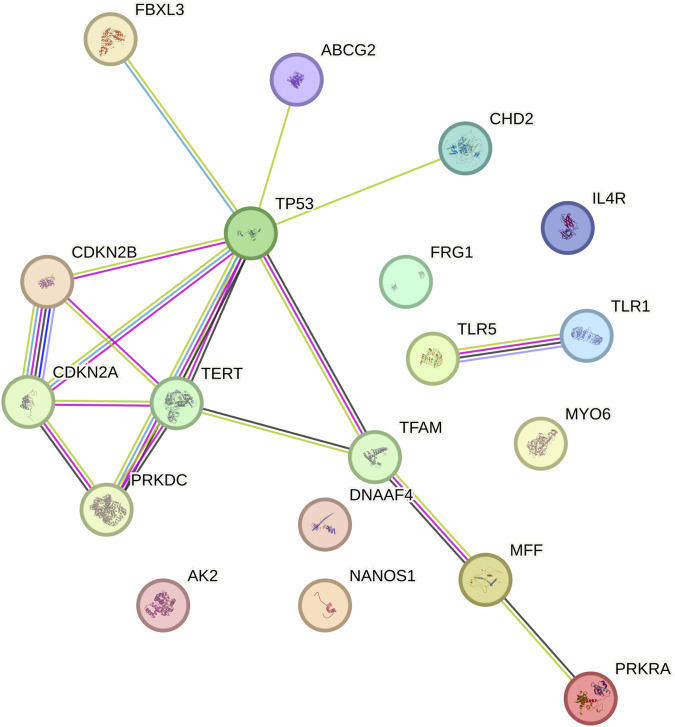
Protein–protein interaction (ppi) network of tumor-specific mutated genes. Protein–protein interaction network constructed using STRING v12 for 21 genes harboring exclusively tumor-specific mutations. The network comprises 19 nodes and 17 edges, significantly exceeding the expected number of interactions (p < 0.00847). Central nodes include TP53-associated interacting genes, suggesting functional clustering within cancer-related and cell cycle regulatory pathways. Nodes represent proteins, and edges represent predicted or experimentally validated interactions.

A protein–protein interaction (PPI) network of 21 genes with exclusively tumor specific mutations in at least two recruited patients was constructed to elucidate their interactions using STRING v12 (Figure 6). The network comprised of 19 nodes and 17 edges. The expected number of edges was 9. This indicates that our network has significantly more interactions than expected (p-value <0.00847), suggesting close interactions among these genes in various cancer related and TP53 associated biological pathways. Notably, AK2, NANOS1, DNAAF4, FRG1, IL4R, and MYO6 were not part of the PPI network.

### 3.9 Polygenic risk score (PRS) estimation

**FIGURE 7:**
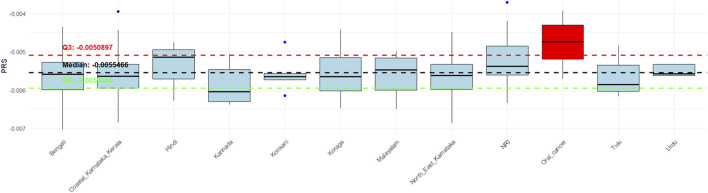
Distribution of polygenic risk scores (PRS) Across Study Participants. Box plot comparing polygenic risk scores (PRS) among OSCC patients and reference South Asian individuals. The lowest PRS quartile (Q1) was used as the reference group. Fifteen of 21 OSCC patients were classified in the highest-risk quartile (Q4), indicating strong inherited susceptibility. Median PRS and interquartile ranges are shown. Statistical comparisons between quartiles were performed using Chi-square analysis.

The study participants, comprising OSCC patients and South Asian individuals from our lab database, were categorized into polygenic risk score (PRS) quartiles (Choi et al., 2019) using the lowest quartile (bottom 25%) as the reference group. All higher PRS quartiles were associated with an elevated risk of developing oral cancer relative to this reference (Q1 vs. Q2: Chi Square = 2.04, p-value = 0.15; Q1 vs. Q3: Chi Square = 4.15, p-value = 0.042; Q1 vs. Q4: Chi Square = 17.6, p-value = 2.72 x 10^−5^). The median PRS was −0.0055362, with an interquartile range (IQR) from −0.00595 to −0.0050734. Strikingly, 15 of the 21 OSCC patients were classified in the highest PRS quartile (Q4), suggesting a strong genetic predisposition to oral cancer (Figure 7). Of the remaining patients, four were in quartile 3 (Q3), and two were in quartile 2 (Q2). These findings underscore the potential of PRS as a valuable tool for cancer risk prediction.

### 3.10 Association of frequently mutated genes in OSCC with nuclear hormone receptors

A considerable subset of recurrently altered genes in OSCC are influenced by nuclear hormone receptor signaling, particularly pathways governed by the Vitamin D receptor (VDR), estrogen receptor (ER), and androgen receptor (AR). These receptors are critical regulators of epithelial cell differentiation, proliferation, and immune modulation, and their dysregulation has been linked to malignant transformation and tumor progression. To investigate potential mechanistic and therapeutic relevance, we evaluated whether the commonly mutated genes in our cohort are connected to these hormonal regulatory networks. The genes exhibiting consistent variation across all OSCC samples are summarized in Table 6, while the involvement of these genes in hormone receptor–mediated pathways is presented in Table 7.

**TABLE 6:** Summary of gene variation patterns identified in OSCC samples.

Parameter	Count
Total number of OSCC samples analyzed	19
Number of genes showing variation/mutation in ≥80% of samples	56
Number of genes showing variation/mutation in 100% of samples	21

Genes with 100% prevalence (N = 21) were considered for subsequent association analysis with nuclear hormone receptors.

**TABLE 7:** Summary of genes exhibiting 100% variation in OSCC samples.

Genes associated	Number	Percentage
Genes associated to all three NRs	7	30%
Genes associated to only VDR and ER, No AR	3	7%
Genes associated to only AR and ER, No VDR	4	5.20%
Genes associated to only AR and VDR, No ER	Nil	​
Genes associated to VDR alone	Nil	​
Genes associated to ER alone	1	​
Genes associated to only AR	1	0.47%
Genes associated to both VDR and ER (with or without AR)	10	49.50%
Total no of the genes linked to any/or all of the NRS	16	76%

Abbreviations used: NRs- Nuclear hormone receptors; VDR-Vitamin D receptor; ER: estrogen receptor; AR-Androgen receptor. Association with NRs, were checked only for genes with 100% prevalence.

We identified multiples genes that exhibited variations in all OSCC samples and have been documented to have associations with nuclear hormone receptor signaling (Table 8). Notably, several of these genes including *ABCB1, CD44, CHI3L1, COMT, NAT2, NOS3, PADI2, TLR1, VKORC1, CYP19A1, CYP2D6, FKBP5, HMGCR, BGLAP, ESR1, CYP2B6, CYP3A5* and *XBP1* — are linked to both Vitamin D receptor (VDR) and estrogen receptor (ER) pathways, highlighting the relevance of these hormonal axes in OSCC biology. Interestingly, all genes associated with VDR signaling also showed connections to ER regulation, and a subset further exhibited interactions with androgen receptor (AR) pathways. Given the established role of ER activation in promoting tumor progression and the antagonistic regulatory influence of VDR on ER signaling, these observations reinforce the importance of hormonal crosstalk in shaping oral carcinogenesis (Supplementary Table S3).

**TABLE 8:** Genes harboring clinically relevant mutations in 100% of OSCC patients with functional roles and nuclear hormone receptor associations.

Sl. No.	Gene	Frequency	Prevalence (%)	Role in cancer	VDR	ER	AR	NR association category
1	ABCB1	19	100	Multidrug resistance	Yes	Yes	Yes	All three NRs
2	CAPN10	19	100	-	No	No	No	No known association
3	CD44	19	100	Tumor metastasis	Yes	Yes	Yes	All three NRs
4	CDC27	19	100	Tumor suppressor/oncogene	No	No	Yes	AR only
5	CHI3L1	19	100	Angiogenesis, proliferation, survival	Yes	Yes	Yes	All three NRs
6	CNN2	19	100	Regulates EGR1 expression	No	Yes	Yes	AR & ER only
7	COMT	19	100	Estrogen metabolism	Yes	Yes	-	VDR & ER only
8	CTBP2	19	100	Corepressor of ER and AR	No	Yes	Yes	AR & ER only
9	IGSF3	19	100	-	No	No	No	No known association
10	IL6	19	100	Anti-tumor immune response	Yes	Yes	Yes	All three NRs
11	IL6-AS1	19	100	-	No	No	No	No known association
12	MAPKAPK3	19	100	Tumor suppressive and promoting	No	Yes	No	ER only
13	MGST3	19	100	-	No	No	No	No known association
14	NAT2	19	100	Metabolizing carcinogens	Yes	Yes	Yes	All three NRs
15	NOS3	19	100	Angiogenesis and drug response	Yes	Yes	Yes	All three NRs
16	OPRM1	19	100	Opioid receptor	No	Yes	Yes	AR & ER only
17	PADI2	19	100	Chemoradiotherapy resistance	Yes	Yes	Yes	All three NRs
18	SLC2A9	19	100	Tumor suppression	No	Yes	Yes	AR & ER only
19	TBXT	19	100	-	No	No	No	No known association
20	TLR1	19	100	Tumor suppressive/promoting	Yes	Yes	No	VDR & ER only
21	VKORC1	19	100	Ferroptosis repressor	Yes	Yes	No	VDR & ER only

Abbreviation used: NRs: Nuclear hormone receptors; VDR: Vitamin D receptor; ER: estrogen receptor; AR: androgen receptor.

These findings further emphasize the need to investigate the functional implications of these variants in the context of the diverse environmental exposures and genetic backgrounds observed in the Indian population. Comprehensive characterization of how such hormone-associated molecular alterations influence tumor behavior may facilitate the discovery of actionable biomarkers and therapeutic targets. Our study supports the rationale for evaluating personalized treatment approaches that integrate vitamin D supplementation or hormone pathway modulators alongside conventional therapies to improve OSCC clinical outcomes.

## 4 Discussion

The study analyzed the mutational landscape of oral squamous cell carcinoma (OSCC) in 21 OSCC patients from Southwest coast of India, revealing significant variation in the total number of clinically relevant mutations per patient. Intronic variants were most prevalent (56%), followed by missense variants (12%) and non-coding transcript variants (10%). ClinVar classification system (SNVs and DELINs). Somatic and germline mutations were identified by comparing tumour tissues with adjacent normal tissues. Variants designated with classifiers such as pathogenic, likely pathogenic, risk allele, drug response, association, likely association, and protective were used for downstream analysis. We found discernible variation among the 21 recruited patients in terms of the total number of clinically relevant mutations identified.56 genes harbored clinically relevant mutations in at least 80% of patients, with 21 of these genes (including *ABCB1, TLR1,* and *VKORC1*) mutated in all patients. Among them *CD44, CDC27, COMT, IL6,* and 16 others were identified in all recruited patients. Among 69 clinically relevant mutations identified in at least 80% of patients, 19 were present in all patients. Novel mutations in *TBXT* and *PERM1*, along with previously reported mutations in *XRCC1* and *ITPKB*, highlight the importance of understanding the local mutational landscape. Gene ontology analysis of the 21 universally mutated genes revealed enrichment in biological processes related to B cell lineage commitment, senescence, and cell cycle regulation, as well as cancer-related pathways like p53 signaling and platinum drug resistance. Protein-protein interaction network analysis of these 21 genes showed a significant enrichment of interactions, suggesting close functional relationships within cancer-related and TP53-associated pathways (Patel et al., 2021), although some genes (*AK2, NANOS1, DNAAF4, FRG1, IL4R,* and *MYO6*) were not integrated into the network.

Our finding of the *TLR1* rs5743618 variant in 100% of our OSCC tumor samples is interesting. While previous studies have investigated *TLR1* in OSCC, a 100% prevalence of this specific SNP has, to our knowledge, not been reported (Rusanen et al., 2024). examined *TLR1* expression in OSCC and found downregulation of TLRs and NF-κB in OSCC, and upregulation of *TLR4* expression with presence of *Candida*., but they did not investigate specific SNPs. This difference in methodology and/or population specific variation might explain why we observed such a high prevalence of some of the variant. Sharma et al. also explored *TLR1* in OSCC and reported progression and suppression of OSCC is associated with different TLRs promoting tumor development and also inhibiting the progression of oral neoplasm (Sharma et al., 2021). Merging research within the Indian context of OSCC has begun to unveil novel genetic and epigenetic alterations that may hold unique significance for this population, where the disease burden is substantial. Studies investigating Toll-like receptor (TLR) polymorphisms have identified specific variants, such as the *TLR9* rs187084 CC genotype, associated with poorer prognostic outcomes in Indian patients (Bharadwaj et al., 2019; Soni et al., 2023) suggesting their potential as population-specific prognostic markers. Furthermore, high-throughput sequencing efforts have pinpointed novel mutations in genes like *CHD8, ITPKB,* and *HNF1A* (Paul et al., 2025), as well as the significant upregulation of *UBE2D1* (Soni et al., 2023), indicating potentially unique pathways driving OSCC in this population and offering novel therapeutic avenues. Epigenetic studies have also highlighted the frequent hypermethylation of tumor suppressor genes like LATS1, with links to prevalent environmental factors such as tobacco use (a study in North Indian population), suggesting their utility as diagnostic biomarkers (Goel et al., 2021). These findings underscore the importance of investigating the distinct genetic and environmental landscape of OSCC in India to identify population-specific risk factors, understand disease heterogeneity, and ultimately pave the way for more tailored diagnostic and therapeutic strategies. This discrepancy could be due to differences in sample size, ethnicity of the studied populations. This highlights the importance of investigating genetic variations at the SNP level to gain a deeper understanding of TLR1’s role in OSCC (Bharadwaj et al., 2019).

X-ray cross-complementing group 1 (*XRCC1*) is expressed in nearly 80% of OSCC tumors and is essential for repairing DNA single-strand breaks through its interactions with DNA ligase III, DNA polymerase β (β-Pol), and poly (ADP-ribose) polymerase (PARP) (London, 2015). Elevated levels of *XRCC1* have been associated with poorer prognosis and limited treatment effectiveness in cancer patients. Moreover, *XRCC1* genetic variants exhibit varied clinical significance—while certain alleles are linked to improved response in oesophageal cancer, they are also associated with a higher likelihood of treatment-related toxicity in head and neck malignancies (Gong et al., 2021). Due to its central role in DNA repair, *XRCC1* serves as an important pharmacogenomic marker, influencing individual sensitivity to radiotherapy and platinum-based chemotherapies.

In OSCC, *XRCC1* polymorphisms are particularly relevant to cisplatin response, as reduced repair capacity can amplify drug-induced cytotoxicity while simultaneously increasing adverse clinical outcomes. Notably, functionally significant variants such as Arg399Gln (rs25487) and Arg194Trp (rs1799782) impair repair efficiency and have been implicated in cisplatin sensitivity and toxicity profiles (Gong et al., 2021). Among the patients included in the present study, the Arg399Gln (rs25487) variant was identified in approximately 80% of cases. Collectively, these findings highlight *XRCC1* mutation screening as a valuable tool for predicting therapeutic response and tailoring individualized treatment strategies in head and neck cancers.

The presence of rs1805010 (intergenic) and rs546905091 (in *PRKDC*) in 46% of our patients suggests a potential role for these variants in OSCC development, although their precise functional significance remains to be determined. The limited existing literature on these specific variants in OSCC makes it difficult to draw direct comparisons. However, *PRKDC* has been implicated in other cancers suggesting a possible role in cell proliferation and apoptosis. Our findings warrant further investigation to explore the functional impact of these variants and their clinical relevance in OSCC.

The polygenic risk score (PRS) analysis presented in Figure 7 reveals significant stratification of genetic predisposition to oral cancer across different South Asian populations. Notably, the majority of oral squamous cell carcinoma (OSCC) patients (16 out of 21) fall into the highest PRS quartile (Q4), reinforcing a strong association between elevated PRS and increased oral cancer risk. This clustering above the 75th percentile suggests that individuals in Q4 have a heightened genetic susceptibility towards oral cancer. Additionally, populations such as the NRI and Hindi groups also show median PRS values approaching or above the upper quartile threshold, indicating elevated genetic risk. In contrast, groups like Tulu, Kannada, and Konkani predominantly lie below the median and lower quartile, suggesting comparatively lower genetic risk of oral cancer (Lee et al., 2024; Chande et al., 2022). These observations support the utility of PRS as a predictive biomarker and reinforce the need for population-specific risk profiling. Incorporating PRS into screening strategies could enable early intervention and personalized prevention in high-risk groups (Lewis and Vassos, 2020).

Additionally, 14 out of 19 genes that have at least one mutation among recruited OSCC patients, are associated with Vitamin D, Estrogen (E2), an Androgen/testosterone receptor signaling (Supplementary Table S1). Among the identified polymorphisms, the genes such as *ABCB1, CD44, CHI3L1, COMT, NAT2, NOS3, PADI2, TLR1, VKORC1, CYP19A1, CYP2D6, FKBP5, HMGCR, BGLAP, ESR1, CYP2B6, CYP3A5, XBP1*, were associated with both Vitamin D and E2 signaling, emphasizing the need to probe into these pathways in context of oral cancers. Interestingly all the 10 genes associated to VDR has associations to ER, and 7 of these genes have known associations with AR as well. Among them ABCB1 is known to be associated with multi drug resistance (Supplementary Table S1). *CD44, CHI3L1, CNN2, CTBP2, NAT2, SLC2A9, TLR1* and *VKORC1* are associated with bladder, prostate, and breast cancers respectively. It is well established that ER is an environmentally regulated gene and promotes cancer progression and VDRs antagonize the action of ERs. There are several genes in cancers with well-established cross talks between ERs and VDRs. To the best of our knowledge this is the first study from India that identified genes associated with OSCC, having link to nuclear hormone receptors. The findings highlight the importance of studying diverse populations to uncover unique mutation patterns influenced by environmental and genetic factors.

Our comprehensive molecular profiling of OSCC patients in a South Indian population revealed several novel mutations, expanding our understanding of the genetic landscape of this disease. Notably, we identified specific genes, e.g., *CD44, CDC27, COMT, IL6*, as harboring previously unreported mutations in this cohort. The identification of these novel mutations raises the possibility that they may also contribute to the development of other cancers. For instance, *PRKDC* mutations have been implicated in other cancer types and could potentially serve as a shared oncogenic driver across different malignancies. Further investigations, including functional studies and large-scale genomic analyses, are needed to determine the prevalence and functional significance of these mutations in other cancer types.

Regional genomic characterization is essential for understanding disease heterogeneity in OSCC, as mutational landscapes are profoundly shaped by environmental exposures, lifestyle behaviors, and ancestry-linked genetic backgrounds. Comparative analysis with the TCGA-HNSCC dataset further underscores this regional specificity. While TCGA-HNSCC predominantly reports high-frequency somatic alterations in canonical oncogenic drivers and tumor suppressors such as *TP53, FAT1, NOTCH1, CASP8,* and *PIK3CA*, our cohort demonstrated a contrasting enrichment of variants in genes involved in immune modulation, xenobiotic metabolism, and host–environment interactions, including *TLR1, NOS3, PADI2, NAT2, *and *VKORC1*, with comparatively fewer disruptions in classical driver pathways. The universal presence of *TLR1*rs5743618 in our cohort, rarely observed at comparable frequency in TCGA-HNSCC, suggests a population-enriched immune-genetic signature that may reflect tumorigenic mechanisms driven by chronic inflammatory exposure, smokeless tobacco use, dietary practices, and region-specific microbial interactions characteristic of coastal Karnataka. Consistent with this observation, systematic TCGA queries restricted to tumor-specific somatic variants revealed either very low or an absence of corresponding mutations for these genes, reinforcing the likelihood that many alterations identified in our cohort represent germline or population-enriched variants rather than recurrent somatic drivers captured in large Western datasets.

A patient-wise comparison with the most frequently mutated TCGA-HNSCC genes further demonstrated limited overlap between the two datasets. Among canonical TCGA-HNSCC drivers, *TP53* was detected in five of 21 patients, *CDKN2A*in three patients, and *EGFR*, a gene commonly altered through copy-number amplification in TCGA, was identified in a single case, whereas several other hallmark TCGA-HNSCC genes, including *FAT1, NOTCH1, KMT2D, NSD1, CASP8, *and *HRAS*, were not observed. Importantly, the regional specificity of this mutational profile is further supported by our previous study (Paul et al., 2025), in which variants in *ITPKB* and *XRCC1* were identified in over 80% of patients, underscoring their consistent enrichment and potential population-specific relevance within the local OSCC cohort.

Collectively, these findings highlight a restricted concordance with the TCGA mutational spectrum and emphasize the presence of a distinct, regionally shaped genomic architecture in this OSCC population. From a clinical perspective, immune-associated polymorphisms may influence carcinogen metabolism, inflammatory signaling, tumor immune evasion, and responsiveness to chemoradiotherapy, while the high prevalence of pharmacogenomic variants in *ABCB1, XRCC1,* and *COMT* may modulate drug transport, DNA repair efficiency, treatment tolerance, and therapeutic resistance. Together, these observations underscore the necessity of adopting contextualized precision oncology strategies tailored to the Indian OSCC population, rather than relying exclusively on biomarker frameworks derived from predominantly Western cohorts.

Overall, the identification of multiple recurrent mutations distinctive to a patient cohort from Southwest coast of Karnataka emphasizes the value of studying genetically diverse groups to fully resolve OSCC heterogeneity and uncover novel pathogenic mechanisms. Future research should investigate the association between these region-specific mutations and local risk factors, integrate functional characterization to determine their mechanistic contribution to OSCC progression, and evaluate their utility as biomarkers for early detection, prognosis, or treatment selection. Validation through expanded, multicentric cohorts, ideally coupled with transcriptomic and epigenomic profiling, will be critical to establish the translational significance of these findings and support the development of community-specific personalized therapeutic strategies.

## 5 Conclusion

Our study provides important insights into the genomic architecture of OSCC cases from the Southwest coast of Karnataka, yet several limitations should be considered. The relatively small cohort size may limit broader generalizability, and while whole-exome sequencing offers significant coverage of coding alterations, additional whole-genome approaches could help capture structural and regulatory variants not assessed here. Moreover, functional characterization of the identified mutations is still needed to establish their mechanistic roles in tumor development. Environmental and habitual exposures, key contributors to OSCC etiology, were not comprehensively analyzed in correlation with mutational patterns, and the interpretation of causality remains inherently challenging in genomic association studies.

Despite these limitations, the findings advance current understanding of region-specific genomic signatures in OSCC. The identification of recurrent alterations in genes such as *TLR1, PRKDC,* and *XRCC1*, including variants linked to immune regulation, DNA repair, and chemotherapy responsiveness, highlights their potential translational relevance. These results emphasize the need to develop population-tailored biomarker panels and personalized therapeutic strategies, particularly in high-burden regions like coastal Karnataka. Future studies incorporating larger, demographically diverse cohorts and functional validation will be essential to translate these discoveries into clinically actionable interventions and improve precision oncology outcomes for OSCC patients.

## Data Availability

The genomic data of the participants cannot be made publicly available due to ethical restrictions. The processed data and summary statistics are available upon request to the first/corresponding authors.

## Supplementary material

SUPPLEMENTARY TABLE S2 Data employed in the stratified analyses. Key clinical and demographic dimensions such as age, sex, primary tumor site and pathological TNM stage were included.

SUPPLEMENTARY TABLE S3 Sex hormone receptor–associated mutated genes identified in OSCC patients from the Southwest Coast of Karnataka. Mutated genes associated with vitamin D receptor (VDR), estrogen receptor (ER), and androgen/testosterone receptor (AR) pathways and previously reported in cancers such as bladder, prostate, and breast cancer.
