# Supplementary material for: Genomic landscape of oral squamous cell carcinoma from the southwest coast of Karnataka: insights from FFPE-based next-generation sequencing
Source: Front Genet. 2026 Feb 25;17:1739925. doi: 10.3389/fgene.2026.1739925 (PMC12975139; doi:10.3389/fgene.2026.1739925)
Supplement: Supplementary file 3 [file Table1.docx]

**Supplementary table 1**: Clinically relevant mutations found in at least 50% recruited patients.

| **Mutation_ID** | **No. of patients with tumor samples (N=19)** | **Chr** | **Position** | **Gene** | **Variant Type** | **Variant Class** | **ClinVar_CLNSIG** | **Codons** | **Amino_acids** | **Mutation type (based on comparing the nearby normal tissue)** |
| --- | --- | --- | --- | --- | --- | --- | --- | --- | --- | --- |
| rs1211152 | 19 | 7 | 87215114 | *ABCB1* | SNV | intron_variant | drug_response |  |  | Germline |
| rs165728 | 19 | 22 | 19957023 | *COMT* | SNV | regulatory_region_variant,3_prime_UTR_variant | drug_response |  |  | Germline |
| rs1800795 | 19 | 7 | 22766645 | *IL6AS1 IL6* | SNV | intron_variant,non_coding_transcript_variant,upstream_gene_variant,regulatory_region_variant | other\|risk_factor |  |  | Germline |
| rs2075572 | 19 | 6 | 154412004 | *OPRM1* | SNV | intron_variant,intron_variant,non_coding_transcript_variant | drug_response |  |  | Germline |
| rs2214102 | 19 | 7 | 87229501 | *ABCB1* | SNV | synonymous_variant,5_prime_UTR_variant,regulatory_region_variant | drug_response | ggA/ggG, | G, | Germline |
| rs2359612 | 19 | 16 | 31103796 | *VKORC1* | SNV | intron_variant | drug_response |  |  | Germline |
| rs540825 | 19 | 6 | 154414446 | *OPRM1* | SNV | intron_variant,non_coding_transcript_variant,intron_variant,missense_variant | drug_response | ,caA/caT | ,Q/H | Germline |
| rs562859 | 19 | 6 | 154414573 | *OPRM1* | SNV | intron_variant,intron_variant,non_coding_transcript_variant,synonymous_variant | drug_response | ,Ctg/Ttg | ,L | Germline |
| rs61730489 | 19 | 1 | 117142868 | *IGSF3* | SNV | stop_gained | Pathogenic | tGg/tAg | W/* | Germline |
| rs6449213 | 19 | 4 | 9994215 | *SLC2A9* | SNV | intron_variant | association |  |  | Germline |
| rs650245 | 19 | 6 | 154428702 | *OPRM1* | SNV | intron_variant,intron_variant,non_coding_transcript_variant,3_prime_UTR_variant | drug_response |  |  | Germline |
| rs675026 | 19 | 6 | 154414563 | *OPRM1* | SNV | intron_variant,intron_variant,non_coding_transcript_variant,synonymous_variant | drug_response | ,ggA/ggG | ,G | Germline |
| rs71021021 | 19 | 6 | 154429085 | *OPRM1* | DEL | intron_variant,non_coding_transcript_exon_variant,intron_variant,non_coding_transcript_variant,3_prime_UTR_variant | drug_response |  |  | Germline |
| rs76203768 | 19 | 10 | 126683151 | *CTBP2* | SNV | missense_variant | Pathogenic | Gtg/Atg | V/M | Germline |
| rs775321736 | 19 | 17 | 45214654 | *CDC27* | SNV | non_coding_transcript_exon_variant,missense_variant | Pathogenic | ,Gct/Act | ,A/T | Germline |
| rs77830704 | 19 | 19 | 1037756 | *CNN2* | SNV | missense_variant,regulatory_region_variant | Pathogenic | Ggc/Agc, | G/S, | Germline |
| rs78386506 | 19 | 19 | 1037766 | *CNN2* | SNV | regulatory_region_variant,missense_variant | Pathogenic | ,cGg/cAg | ,R/Q | Germline |
| rs9282821 | 19 | 6 | 154414375 | *OPRM1* | SNV | intron_variant,intron_variant,non_coding_transcript_variant | drug_response |  |  | Germline |
| rs9333378 | 19 | 1 | 165601466 | *MGST3* | SNV | TF_binding_site_variant,intron_variant,regulatory_region_variant | association |  |  | Germline |
| rs1005753 | 18 | 1 | 17444769 | *PADI2* | SNV | regulatory_region_variant,intron_variant | association |  |  | Germline |
| rs1016793 | 18 | 7 | 87199182 | *ABCB1* | SNV | intron_variant | drug_response |  |  | Germline |
| rs11337008 | 18 | 16 | 70896015 | *HYDIN* | DEL | frameshift_variant | Likely_pathogenic | atT/at | I/X | Germline |
| rs1202168 | 18 | 7 | 87195962 | *ABCB1* | SNV | intron_variant | drug_response |  |  | Germline |
| rs1202170 | 18 | 7 | 87195106 | *ABCB1* | SNV | intron_variant | drug_response |  |  | Germline |
| rs12460842 | 18 | 19 | 10222195 | *PPANP2RY11 PPAN P2RY11* | SNV | intron_variant,3_prime_UTR_variant,upstream_gene_variant,regulatory_region_variant | association |  |  | Germline |
| rs12654264 | 18 | 5 | 74648603 | *HMGCR* | SNV | intron_variant | association |  |  | Germline |
| rs1551570 | 18 | 19 | 10218030 | *PPANP2RY11 PPAN* | SNV | intron_variant,regulatory_region_variant | association |  |  | Germline |
| rs1670533 | 18 | 4 | 1078187 | *RNF212* | SNV | intron_variant,intron_variant,non_coding_transcript_variant | association |  |  | Germline |
| rs2070744 | 18 | 7 | 150690079 | *NOS3* | SNV | intron_variant,regulatory_region_variant | protective |  |  | Germline |
| rs3127334 | 18 | 6 | 166574246 | *TBXT* | SNV | intron_variant | risk_factor |  |  | Germline |
| rs3826784 | 18 | 19 | 10227010 | *EIF3G* | SNV | intron_variant | association |  |  | Germline |
| rs414171 | 18 | 3 | 50649499 | *MAPKAPK3* | SNV | 5_prime_UTR_variant,regulatory_region_variant | risk_factor |  |  | Germline |
| rs4713916 | 18 | 6 | 35669983 | *FKBP5* | SNV | intron_variant | Likely_risk_allele |  |  | Germline |
| rs4950928 | 18 | 1 | 203155882 | *CHI3L1* | SNV | upstream_gene_variant,regulatory_region_variant | risk_factor |  |  | Germline |
| rs5743618 | 18 | 4 | 38798648 | *TLR1* | SNV | missense_variant | Uncertain_risk_allele\|protective | aGc/aTc | S/I | Somatic |
| rs708776 | 18 | 1 | 226923505 | *ITPKB* | SNV | missense_variant,regulatory_region_variant | Likely_pathogenic | cCg/cAg, | P/Q, | Germline |
| rs727479 | 18 | 15 | 51534547 | *CYP19A1 MIR4713 MIR4713HG* | SNV | intron_variant,downstream_gene_variant,intron_variant,non_coding_transcript_variant | drug_response |  |  | Germline |
| rs7417106 | 18 | 1 | 911595 | *PERM1* | SNV | TF_binding_site_variant,missense_variant,regulatory_region_variant | Pathogenic | ,gTg/gCg | ,V/A | Germline |
| rs10789501 | 17 | 1 | 47609489 | *CYP4A22* | SNV | missense_variant,intron_variant,regulatory_region_variant | association | Tgt/Cgt, | C/R, | Germline |
| rs1333042 | 17 | 9 | 22103813 | *CDKN2BAS1* | SNV | intron_variant,non_coding_transcript_variant,regulatory_region_variant | protective |  |  | Germline |
| rs1360780 | 17 | 6 | 35607571 | *FKBP5* | SNV | intron_variant | Likely_risk_allele |  |  | Germline |
| rs1801253 | 17 | 10 | 115805056 | *ADRB1* | SNV | missense_variant,regulatory_region_variant | association\|drug_response | Gga/Cga, | G/R, | Germline |
| rs2124437 | 17 | 2 | 33682737 | *RASGRP3* | SNV | intron_variant,regulatory_region_variant | association |  |  | Germline |
| rs2142697 | 17 | 20 | 7906943 | *HAO1* | SNV | regulatory_region_variant,intron_variant | association |  |  | Germline |
| rs2305795 | 17 | 19 | 10226052 | *PPANP2RY11 P2RY11 EIF3G* | SNV | downstream_gene_variant,intron_variant | association |  |  | Germline |
| rs2423326 | 17 | 20 | 7893640 | *HAO1* | SNV | intron_variant,regulatory_region_variant | association |  |  | Germline |
| rs2488457 | 17 | 1 | 114415368 | *AP4B1AS1* | SNV | intron_variant,non_coding_transcript_variant,regulatory_region_variant | risk_factor |  |  | Germline |
| rs28371730 | 17 | 22 | 42523209 | *CYP2D6* | SNV | intron_variant | drug_response |  |  | Germline |
| rs3796619 | 17 | 4 | 1095281 | *RNF212* | SNV | intron_variant,intron_variant,non_coding_transcript_variant | association |  |  | Germline |
| rs497332 | 17 | 6 | 154431393 | *OPRM1* | SNV | intron_variant,intron_variant,non_coding_transcript_variant | drug_response |  |  | Germline |
| rs6118004 | 17 | 20 | 7897049 | *HAO1* | SNV | intron_variant | association |  |  | Germline |
| rs6667260 | 17 | 1 | 226923938 | *ITPKB* | SNV | missense_variant,regulatory_region_variant | Likely_pathogenic | Tcc/Gcc, | S/A, | Germline |
| rs759330 | 17 | 1 | 156213257 | *PAQR6 PMF1BGLAP BGLAP* | SNV | 3_prime_UTR_variant,downstream_gene_variant,non_coding_transcript_exon_variant | association |  |  | Germline |
| rs1008438 | 16 | 6 | 31783208 | *HSPA1A HSPA1L* | SNV | upstream_gene_variant,regulatory_region_variant,TF_binding_site_variant | association |  |  | Germline |
| rs1063192 | 16 | 9 | 22003367 | *CDKN2BAS1 CDKN2B* | SNV | intron_variant,non_coding_transcript_variant,3_prime_UTR_variant | protective |  |  | Germline |
| rs10954213 | 16 | 7 | 128589427 | *IRF5* | SNV | 3_prime_UTR_variant | risk_factor |  |  | Germline |
| rs111033566 | 16 | 7 | 142458451 | *PRSS1* | SNV | missense_variant | Pathogenic | aAc/aTc | N/I | Germline |
| rs12647681 | 16 | 4 | 69963160 | *UGT2B7* | SNV | intron_variant | drug_response |  |  | Germline |
| rs12647682 | 16 | 4 | 69963168 | *UGT2B7* | SNV | intron_variant | drug_response |  |  | Germline |
| rs165599 | 16 | 22 | 19956781 | *COMT* | SNV | 3_prime_UTR_variant | drug_response |  |  | Germline |
| rs1799930 | 16 | 8 | 18258103 | *NAT2* | SNV | missense_variant | drug_response | cGa/cAa | R/Q | Germline |
| rs2004640 | 16 | 7 | 128578301 | *IRF5* | SNV | regulatory_region_variant,splice_donor_variant,intron_variant | Pathogenic\|risk_factor |  |  | Germline |
| rs20541 | 16 | 5 | 131995964 | *IL13* | SNV | missense_variant | risk_factor | cAg/cGg | Q/R | Germline |
| rs2070634 | 16 | 3 | 186336027 | *AHSG* | SNV | intron_variant | association |  |  | Germline |
| rs3766379 | 16 | 1 | 160807715 | *CD244* | SNV | regulatory_region_variant,intron_variant | risk_factor |  |  | Germline |
| rs4292394 | 16 | 4 | 69972949 | *UGT2B7* | SNV | synonymous_variant | drug_response | ctC/ctG | L | Germline |
| rs7294 | 16 | 16 | 31102321 | *VKORC1* | SNV | regulatory_region_variant,3_prime_UTR_variant | drug_response |  |  | Germline |
| rs7439366 | 16 | 4 | 69964338 | *UGT2B7* | SNV | missense_variant,regulatory_region_variant,TF_binding_site_variant | drug_response | Tat/Cat, | Y/H, | Germline |
| rs7668258 | 16 | 4 | 69962078 | *UGT2B7* | SNV | upstream_gene_variant,intron_variant,regulatory_region_variant,TF_binding_site_variant | drug_response |  |  | Germline |
| rs1053874 | 15 | 16 | 3707747 | *DNASE1* | SNV | missense_variant,non_coding_transcript_exon_variant | risk_factor | cGa/cAa, | R/Q, | Germline |
| rs10877012 | 15 | 12 | 58162085 | *METTL1* | SNV | downstream_gene_variant | Likely_risk_allele |  |  | Germline |
| rs112762 | 15 | 11 | 35192629 | *CD44* | SNV | intron_variant | association |  |  | Germline |
| rs12169962 | 15 | 22 | 42522312 | *CYP2D6* | SNV | downstream_gene_variant | drug_response |  |  | Germline |
| rs1341667 | 15 | 10 | 70641860 | *STOX1* | SNV | missense_variant | risk_factor | Tac/Cac | Y/H | Germline |
| rs2235020 | 15 | 7 | 87199265 | *ABCB1* | SNV | intron_variant | drug_response |  |  | Germline |
| rs2235021 | 15 | 7 | 87199264 | *ABCB1* | SNV | intron_variant | drug_response |  |  | Germline |
| rs25487 | 15 | 19 | 44055726 | *XRCC1* | SNV | missense_variant | drug_response | cAg/cGg | Q/R | Germline |
| rs4833095 | 15 | 4 | 38799710 | *TLR1* | SNV | missense_variant | risk_factor | aAt/aGt | N/S | Germline |
| rs7291467 | 15 | 22 | 37972628 | *LGALS2* | SNV | intron_variant | risk_factor |  |  | Germline |
| rs7438244 | 15 | 4 | 69964209 | *UGT2B7* | SNV | TF_binding_site_variant,regulatory_region_variant,intron_variant | drug_response |  |  | Germline |
| rs7557402 | 15 | 2 | 46603671 | *EPAS1* | SNV | splice_region_variant,intron_variant | drug_response |  |  | Germline |
| rs1051266 | 14 | 21 | 46957794 | *SLC19A1* | SNV | missense_variant,5_prime_UTR_variant | drug_response | cAc/cGc, | H/R, | Germline |
| rs1748033 | 14 | 1 | 17662662 | *PADI4* | SNV | TF_binding_site_variant,regulatory_region_variant,synonymous_variant | association | ,Ttg/Ctg | ,L | Germline |
| rs2070635 | 14 | 3 | 186336176 | *AHSG* | SNV | intron_variant | association |  |  | Germline |
| rs2076615 | 14 | 1 | 17413121 | *PADI2* | SNV | synonymous_variant,regulatory_region_variant | association | ggT/ggG, | G, | Germline |
| rs2165241 | 14 | 15 | 74222202 | *LOXL1* | SNV | intron_variant | risk_factor |  |  | Germline |
| rs2305764 | 14 | 19 | 17313833 | *MYO9B* | SNV | intron_variant | risk_factor |  |  | Germline |
| rs353647 | 14 | 11 | 35191445 | *CD44* | SNV | intron_variant | association |  |  | Germline |
| rs4402960 | 14 | 3 | 185511687 | *IGF2BP2* | SNV | regulatory_region_variant,intron_variant,intron_variant,non_coding_transcript_variant | risk_factor |  |  | Germline |
| rs6851533 | 14 | 4 | 69978750 | *UGT2B7* | SNV | downstream_gene_variant | drug_response |  |  | Germline |
| rs7116432 | 14 | 11 | 35244058 | *CD44* | SNV | intron_variant,regulatory_region_variant,3_prime_UTR_variant | association |  |  | Germline |
| rs10757274 | 13 | 9 | 22096055 | *CDKN2BAS1* | SNV | intron_variant,non_coding_transcript_variant | risk_factor |  |  | Germline |
| rs111564371 | 13 | 22 | 42524708 | *CYP2D6* | SNV | intron_variant | drug_response |  |  | Germline |
| rs11203366 | 13 | 1 | 17657534 | *PADI4* | SNV | missense_variant | association | Ggc/Agc | G/S | Germline |
| rs11203367 | 13 | 1 | 17657616 | *PADI4* | SNV | missense_variant | association | gTg/gCg | V/A | Germline |
| rs112568578 | 13 | 22 | 42524713 | *CYP2D6* | SNV | intron_variant | drug_response |  |  | Germline |
| rs113889384 | 13 | 22 | 42524743 | *CYP2D6* | SNV | intron_variant | drug_response |  |  | Germline |
| rs1801275 | 13 | 16 | 27374400 | *IL4R* | SNV | missense_variant | risk_factor | cAg/cGg | Q/R | Germline |
| rs2057094 | 13 | 1 | 17405949 | *PADI2* | SNV | intron_variant,regulatory_region_variant | association |  |  | Germline |
| rs2108622 | 13 | 19 | 15990431 | *CYP4F2* | SNV | missense_variant | drug_response | Gtg/Atg | V/M | Germline |
| rs2234693 | 13 | 6 | 152163335 | *ESR1* | SNV | intron_variant | risk_factor |  |  | Germline |
| rs2240340 | 13 | 1 | 17662639 | *PADI4* | SNV | intron_variant,regulatory_region_variant | association |  |  | Germline |
| rs2269577 | 13 | 22 | 29196757 | *XBP1* | SNV | TF_binding_site_variant,upstream_gene_variant,regulatory_region_variant | risk_factor |  |  | Germline |
| rs3745274 | 13 | 19 | 41512841 | *CYP2B6* | SNV | missense_variant | drug_response | caG/caT | Q/H | Germline |
| rs3842570 | 13 | 2 | 241534241 | *CAPN10* | - | intron_variant | risk_factor |  |  | Germline |
| rs4512367 | 13 | 8 | 68927592 | *PREX2* | SNV | intron_variant,regulatory_region_variant | association |  |  | Germline |
| rs4646450 | 13 | 7 | 99266318 | *CYP3A5 ZSCAN25* | SNV | intron_variant,3_prime_UTR_variant,intron_variant,non_coding_transcript_variant | association |  |  | Germline |
| rs4977574 | 13 | 9 | 22098574 | *CDKN2BAS1* | SNV | intron_variant,non_coding_transcript_variant,regulatory_region_variant | risk_factor |  |  | Germline |
| rs7110737 | 13 | 11 | 35221862 | *CD44* | SNV | intron_variant | association |  |  | Germline |
| rs7794745 | 13 | 7 | 146489606 | *CNTNAP2* | SNV | intron_variant | risk_factor |  |  | Germline |
| rs874881 | 13 | 1 | 17660499 | *PADI4* | SNV | missense_variant | association | gGg/gCg | G/A | Germline |
| rs9406328 | 13 | 6 | 169635010 | *THBS2 THBS2AS1* | SNV | intron_variant,splice_region_variant,intron_variant,intron_variant,non_coding_transcript_variant,splice_region_variant,intron_variant,non_coding_transcript_variant | risk_factor |  |  | Germline |
| rs10246939 | 12 | 7 | 141672604 | *TAS2R38* | SNV | missense_variant | drug_response | Atc/Gtc | I/V | Germline |
| rs1042713 | 12 | 5 | 148206440 | *ADRB2* | SNV | regulatory_region_variant,missense_variant | drug_response | ,Gga/Aga | ,G/R | Germline |
| rs1058164 | 12 | 22 | 42525132 | *CYP2D6* | SNV | synonymous_variant,intron_variant | drug_response | gtC/gtG, | V, | Germline |
| rs10929302 | 12 | 2 | 234665782 | *UGT1A6 UGT1A10 UGT1A8 UGT1A7 UGT1A5 UGT1A3 UGT1A9 UGT1A4* | SNV | intron_variant,regulatory_region_variant,TF_binding_site_variant | drug_response |  |  | Germline |
| rs1143627 | 12 | 2 | 113594387 | *IL1B* | SNV | upstream_gene_variant,regulatory_region_variant | risk_factor |  |  | Germline |
| rs11692304 | 12 | 2 | 103095404 | *SLC9A4* | SNV | regulatory_region_variant,synonymous_variant | association | ,tcG/tcA | ,S | Germline |
| rs11978267 | 12 | 7 | 50466304 | *IKZF1* | SNV | intron_variant,regulatory_region_variant | association |  |  | Germline |
| rs1421085 | 12 | 16 | 53800954 | *FTO* | - | intron_variant,TF_binding_site_variant,regulatory_region_variant,intron_variant,non_coding_transcript_variant | risk_factor |  |  | Germline |
| rs145033890 | 12 | 1 | 38173983 | *CDCA8* | DEL | splice_region_variant,intron_variant | Likely_pathogenic |  |  | Germline |
| rs147889095 | 12 | 1 | 226924875 | *ITPKB* | DEL | inframe_deletion,regulatory_region_variant | Likely_pathogenic | agCAGCGGCAGt/agt, | SSGS/S, | Germline |
| rs17425208 | 12 | 4 | 190876196 | *FRG1* | SNV | missense_variant | Pathogenic | Gcc/Acc | A/T | Germline |
| rs1800566 | 12 | 16 | 69745145 | *NQO1* | SNV | missense_variant,regulatory_region_variant | Pathogenic\|risk_factor | Cct/Tct, | P/S, | Germline |
| rs1985842 | 12 | 22 | 42523409 | *CYP2D6* | SNV | intron_variant | drug_response |  |  | Germline |
| rs20455 | 12 | 6 | 39325078 | *KIF6* | SNV | missense_variant | drug_response | Tgg/Cgg | W/R | Germline |
| rs2242480 | 12 | 7 | 99361466 | *CYP3A4* | SNV | intron_variant | drug_response |  |  | Germline |
| rs28371699 | 12 | 22 | 42526484 | *CYP2D6* | SNV | intron_variant | drug_response |  |  | Germline |
| rs28371702 | 12 | 22 | 42525952 | *CYP2D6* | SNV | intron_variant | drug_response |  |  | Germline |
| rs3812718 | 12 | 2 | 166909544 | *SCN1A* | SNV | intron_variant,non_coding_transcript_variant,intron_variant | drug_response |  |  | Germline |
| rs4337789 | 12 | 4 | 69973044 | *UGT2B7* | SNV | intron_variant | drug_response |  |  | Somatic |
| rs4673993 | 12 | 2 | 216212339 | *ATIC* | SNV | intron_variant | drug_response |  |  | Germline |
| rs713598 | 12 | 7 | 141673345 | *TAS2R38* | SNV | missense_variant | drug_response | Gca/Cca | A/P | Germline |
| rs7439326 | 12 | 4 | 69964180 | *UGT2B7* | SNV | TF_binding_site_variant,intron_variant,regulatory_region_variant | drug_response |  |  | Germline |
| rs10276036 | 11 | 7 | 87180198 | *ABCB1* | SNV | intron_variant | drug_response |  |  | Germline |
| rs11196205 | 11 | 10 | 114807047 | *TCF7L2* | SNV | intron_variant | risk_factor |  |  | Germline |
| rs2235013 | 11 | 7 | 87178626 | *ABCB1* | SNV | intron_variant | drug_response |  |  | Germline |
| rs2235033 | 11 | 7 | 87179143 | *ABCB1* | SNV | intron_variant | drug_response |  |  | Germline |
| rs2235046 | 11 | 7 | 87174066 | *ABCB1* | SNV | intron_variant | drug_response |  |  | Germline |
| rs2274567 | 11 | 1 | 207753621 | *CR1* | SNV | missense_variant | protective | cAt/cGt | H/R | Germline |
| rs4132601 | 11 | 7 | 50470604 | *IKZF1* | - | 3_prime_UTR_variant | association |  |  | Germline |
| rs4646437 | 11 | 7 | 99365083 | *CYP3A4* | SNV | intron_variant | drug_response |  |  | Germline |
| rs546905091 | 11 | 8 | 48844056 | *PRKDC* | INS | intron_variant | Pathogenic |  |  | Somatic |
| rs1006737 | 10 | 12 | 2345295 | *CACNA1C* | - | intron_variant | Uncertain_risk_allele |  |  | Germline |
| rs11276 | 10 | 12 | 14993439 | *ART4* | SNV | missense_variant | Affects | Gac/Aac | D/N | Germline |
| rs12648462 | 10 | 4 | 69963231 | *UGT2B7* | SNV | intron_variant | drug_response |  |  | Germline |
| rs12987977 | 10 | 2 | 102975336 | *IL18R1* | SNV | intron_variant,regulatory_region_variant | association |  |  | Germline |
| rs1667394 | 10 | 15 | 28530182 | *HERC2* | SNV | intron_variant | association |  |  | Germline |
| rs1801280 | 10 | 8 | 18257854 | *NAT2* | SNV | missense_variant | drug_response | aTt/aCt | I/T | Germline |
| rs1805010 | 10 | 16 | 27356203 | *IL4R* | SNV | regulatory_region_variant,5_prime_UTR_variant,missense_variant | Pathogenic\|protective | ,Atc/Gtc | ,I/V | Somatic |
| rs324981 | 10 | 7 | 34818113 | *NPSR1 NPSR1AS1* | SNV | regulatory_region_variant,missense_variant,intron_variant,non_coding_transcript_variant,intron_variant | risk_factor | ,aAt/aTt | ,N/I | Germline |
| rs3749977 | 10 | 6 | 29080344 | *OR2J3* | SNV | missense_variant,regulatory_region_variant | Affects | cGa/cAa, | R/Q, | Germline |
| rs7754840 | 10 | 6 | 20661250 | *CDKAL1* | SNV | regulatory_region_variant,intron_variant | risk_factor |  |  | Germline |
| rs916977 | 10 | 15 | 28513364 | *HERC2* | SNV | intron_variant | Affects |  |  | Germline |
